# Myxome du ventricule droit chez un enfant: une présentation très rare

**DOI:** 10.11604/pamj.2016.25.138.10563

**Published:** 2016-11-04

**Authors:** Amine Tarmiz, Imene Mgarrech, Mehdi Slim, Chokri Kortas, Sofiane Jerbi

**Affiliations:** 1 Service de Chirurgie Cardiovasculaire et Thoracique, CHU Sahloul, Sousse, Tunisie; 2 Service de Cardiologie, CHU Sahloul, Sousse, Tunisie

**Keywords:** Myxome, ventricule droit, embolie pulmonaire, syncope, Myxoma, right ventricle, pulmonary embolism, syncope

## Abstract

Le myxome du ventricule droit est une localisation très rare des myxomes cardiaques. Les complications les plus fréquentes sont l'embolie pulmonaire et l'obstruction par la tumeur de la valve pulmonaire. Nous rapportons le cas d'un enfant âgé de 11 ans admis en Cardiologie pour des syncopes à répétition. L'échographie cardiaque met en évidence un myxome du ventricule droit de 2cm, obstruant l'orifice pulmonaire. L'exérèse chirurgicale est pratiquée en urgence sous circulation extracorporelle, avec des suites opératoires favorables. L'examen anatomopathologique de la pièce a permis de confirmer le diagnostic de myxome. Le suivi à 18 mois ne montre pas de récidive tumorale.

## Abstract (en)

The right ventricle is an uncommon location for cardiac myxoma. Its most common complications are pulmonary embolism and obstruction due to pulmonary valve tumor. We report the case of a 11-year old child with repetitive syncopes admitted to Cardiology ward. Echocardiography showed a right ventricular myxoma of 2cm obstructing the pulmonary orifice. Emergency surgical excision was performed under cardiopulmonary bypasss with favorable postoperative course. Anatomo-pathological examination of the surgical specimen confirmed the diagnosis of myxoma. At 18 months follow-up, there was no evidence of tumor recurrence.

## Introduction

Les myxomes cardiaques sont les tumeurs cardiaques primitives les plus fréquentes [1]. Leur localisation au niveau du ventricule droit (VD) demeure exceptionnelle et intéresse seulement 3% des cas [2]. Nous rapportons le cas d´un myxome du VD, survenant chez un enfant et révélé par des syncopes à répétition.

## Patient et observation

**Figure 1:**
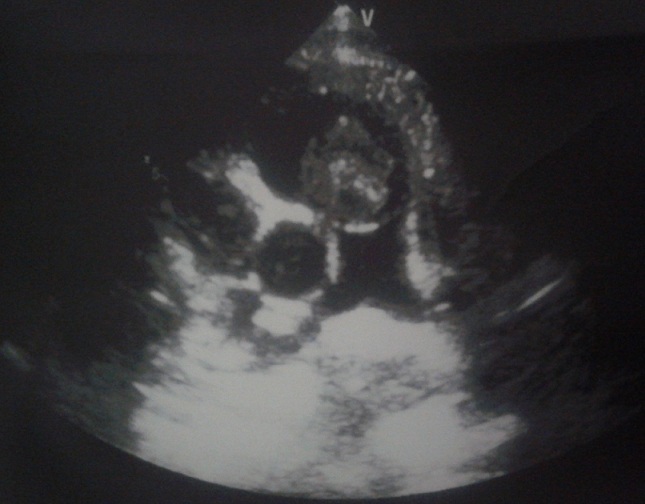
Échographie cardiaque transthoracique montrant une masse arrondie, de 25x20 mm au niveau de l’infundibulum pulmonaire, mobile et obstruant partiellement la chambre de chasse du VD durant la systole

Un enfant de sexe masculin, âgé de 11 ans, est hospitalisé en cardiologie pour des syncopes à répétition évoluant depuis deux semaines. Aucun antécédent pathologique n´a été relevé. À l´examen physique, l´état général est conservé et les constantes vitales sont normales. L´auscultation cardiaque met en évidence un souffle holosystolique, d´intensité 4/6, latéro-sternal gauche, sans irradiation particulière. L´électrocardiogramme montre un rythme régulier sinusal à 82 battements/minutes, sans troubles de repolarisation ni de conduction. La radiographie du thorax est strictement normale. L´échographie cardiaque, en mode transthoracique, objective une masse arrondie de 25 x 20 mm au niveau de l´infundibulum pulmonaire, mobile et obstruant partiellement la chambre de chasse du VD (Figure 1) durant la systole.

**Figure 2:**
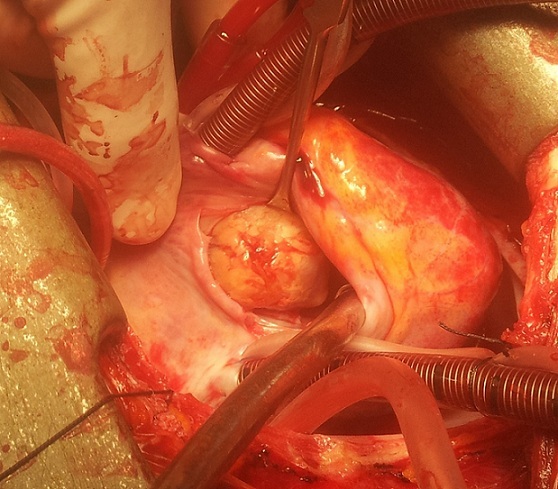
Vue opératoire du myxome à travers une auriculotomie droite

L´enfant est opéré en urgence. La voie d´abord est une sternotomie médiane verticale. Une circulation extracorporelle est mise en place, en normothermie, entre l´aorte ascendante et les deux veines caves. Un abord, via une atriotomie droite, permet de mettre en évidence une masse tumorale sphéroïde d´environ 3 cm de grand axe, qui prend son origine par un pédicule large sur le versant infundibulaire du septum interventriculaire (SIV), à environ 1 cm du plan de la valve pulmonaire (Figure 2). La tumeur est réséquée en bloc, emportant largement sa base d´implantation sur le SIV. La valve pulmonaire est respectée. Le contrôle par échographie cardiaque transoesophagienne peropératoire montre une chambre de chasse du VD libre de toute masse résiduelle, ainsi que la compétence des valves pulmonaire et tricuspide. L´évolution post-opératoire est favorable. L´étude anatomopathologique de la pièce opératoire confirme le diagnostic de myxome. Le suivi échographique à 18 mois post-opératoire ne montre pas de récidive tumorale.

## Discussion

Les myxomes représentent les tumeurs cardiaques les plus fréquentes [1]. Dans la grande majorité des cas, elles sont localisées au niveau des oreillettes, notamment l´oreillette gauche [3] et leur localisation au niveau du ventricule droit est très rare, survenant seulement dans environ 3% des cas [2]. L´âge moyen de révélation de cette pathologie est de 50 ans [4]. La grande majorité des myxomes sont de localisation unique, les localisations multiples se trouvent plus fréquemment dans les formes familiales qui constituent moins de 10% des myxomes [2, 4]. La découverte d´un myxome cardiaque chez un enfant ou un adulte jeune, avec une localisation aussi rare que le ventricule droit, doit absolument faire évoquer une forme familiale ainsi que l´association au Complexe de Carney [5].Ce dernier est une maladie congénitale, à transmission autosomique dominante et à fort potentiel néoplasique, caractérisée par l´association de myxomes cardiaques, souvent multiples, de pigmentations cutanées irrégulières et d´une hyperactivité endocrine [6].

Récemment, des études génétiques [7] ont montré que la présence de mutations du gène PRKAR1A étaient associées aux formes familiales alors que dans les formes sporadiques, aucune mutation n´a été identifiée. Chez notre patient, l´histoire familiale n´a pas montré d´antécédents de myxomes, de tumeurs ou de pigmentations cutanées inhabituelles. S´agissant vraisemblablement d´une forme sporadique et solitaire, aucune étude génétique n´était alors justifiée dans ce cas.

Le myxome du ventricule droit est le plus fréquemment révélé par une embolie pulmonaire. Il existe par ailleurs un risque d’enclavement de la tumeur dans la valve pulmonaire pouvant être responsable de syncope ou de mort subite [2]. Dans notre cas, la tumeur était très mobile et semblait obstruer, en systole, la chambre de chasse du VD, expliquant ainsi les épisodes répétés de syncope chez cet enfant.

Une fois le diagnostic suspecté à l´échographie, la chirurgie doit être entreprise en urgence à cause de la haute fréquence des évènements emboliques (30% des cas) [4]. L’exérèse doit se faire en un bloc afin d’éviter les emboles de fragments tumoraux. L’électrocoagulation de la base d’implantation du myxome et l’excision d’une partie d’endocarde sain semble prévenir la récidive [8]. En cas d’extension à l’artère pulmonaire ou de suspicion d’embolie pulmonaire, une exploration de l’artère pulmonaire doit être réalisée en vue d’une embolectomie [8].

Actuellement, la chirurgie sous circulation extracorporelle demeure le traitement de choix des myxomes cardiaques et les patients opérés bénéficient d´une survie équivalente à celle de la population générale [3]. Néanmoins, le suivi échographique à long terme doit être rigoureux, afin de détecter précocement d´éventuelles récidives, surtout chez les patients jeunes [1].

## Conclusion

Le myxome du VD est une pathologie très rare qui doit faire rechercher une forme familiale telle que le Complexe de Carney, notamment chez l'enfant ou l'adulte jeune. Le traitement repose sur l'exérèse chirurgicale qui doit se faire sans délai afin d'éviter deux complications redoutables: l'embolie pulmonaire et l'obstruction de la valve pulmonaire par la masse tumorale pouvant entrainer une syncope ou une mort subite.
